# Using scenarios to assess the future supply of NHS nursing staff in England

**DOI:** 10.1186/1478-4491-10-16

**Published:** 2012-07-12

**Authors:** James Buchan, Ian Seccombe

**Affiliations:** 1 School of Health Sciences, Queen Margaret University, QMU Drive, Edinburgh, EH 21 6UU, UK

## Abstract

This paper examines issues related to the future supply of registered nursing staff, midwives and health visitors in the National Health Service (NHS) in England at a time when there are major public sector funding constraints and as more of these staff are reaching retirement age. Based on available workforce data, the paper reviews different possible scenarios for the supply of NHS nurses over a ten year period, assessing the impact of different numbers of new staff being trained and of varying retirement patterns from the ageing profession.

The government in England has more policy levers available than is the case in many other countries. It determines the number of pre-registration training places that are commissioned and funded, it is the major employer, and it also controls the inflow of nurses from other countries through migration policies. Scenario models provide a picture of what the future might look like under various assumptions. These outcomes can be quantified and the results used to assess the risks and opportunities of alternate policy decisions. The approach used in this paper is that of the aggregate deterministic supply model.

As part of this exercise, eight scenarios were selected and modelled. These were:

A. “No change”- current inflows and outflows

B. “Redundancies” - current inflow with higher outflow

C. “Improved retention” - current inflow with lower outflow

D. “Reduced training intakes A” - lower inflows with lower outflow

E. “Reduced training intakes B” - lower inflow with higher outflows

F. “Pension time-bomb”- current inflow with a higher rate of retirement

G. “Pension delayed”- current inflow with a lower rate of retirement

H. “Worst case” - lower inflow and higher outflow including higher retirement

Most of the scenarios indicate that a reduction in the supply of nursing staff to NHS England is possible over the next ten years. Small changes in assumptions can make a substantial difference to outcomes and therefore emphasize the point that it is unwise to base policy decisions on a single projection. It is important that different scenarios are considered that may be regarded as possible futures, based on a realistic assessment of the available workforce data, policies and broader labour market and funding outlook.

## Introduction

This paper examines issues related to the future supply of nurses, midwives and health visitors in the National Health Service (NHS) in England at a time when there are major public sector funding constraints and as more nurses are reaching the age they may retire- either voluntarily or when they reach mandatory retirement age. For the purposes of the scenario modelling, the focus is on this broad based workforce; midwives and health visitors are separately identifiable but much smaller components of the overall nursing and midwifery workforce. Based on available workforce data, the paper reviews different possible scenarios for the supply of NHS nursing staff over the period to 2022, assessing the impact of different numbers of new staff being trained and of varying retirement patterns from the ageing profession.

## Background

Nursing staff education and employment is located mainly in the public sector in England, and is, therefore, vulnerable to any policy changes that focus on public sector cost containment. All pre-registration nurse education is publicly funded and the vast majority of working registered nurses, midwives and health visitors are employed in the public sector, mainly in the NHS [1]. Since the global financial crises of 2008/2009 there has been a period of “austerity” measures to contain public sector expenditure in the England [2]. There have been increased concerns about constraints on NHS funding streams [3] and the knock-on effect on the nursing labour market, with increasing reports locally about job freezes, increased workload and reduced staffing, as well as reductions in the inflow of student nurses entering pre-registration nurse education [4].

As well as funding constraints on new supply, a second critical factor that will determine the profile and dynamics of the NHS nursing and midwifery workforce over the next ten years is the ageing of the workforce. Currently about one in three United Kindgom-based nurses and midwives is aged 50 or older [1] and as is the case in other developed countries, many will soon reach possible retirement age. While the pattern of retirement behaviour may be affected by changes in pension provision and normal retirement age, at best this will only delay the withdrawal of these staff from the labour market. Policy makers need to develop a better understanding of the likely impact of nurse and midwife retirement in order to plan how to deal with it.

In assessing the future supply of staff, it is also important to highlight the effect of several critical contextual factors in the NHS in England:

· The impact of the post recession economic situation has meant that the general labour market in England is exhibiting increased unemployment and reduced job mobility. The need to maintain employment and increase income at a difficult economic time has meant that the supply of nursing “hours” has increased, as more nurses attempt to re-enter the nursing labour market or increase their hours of work, while others delay retirement [1];

· The demand for NHS services is expected to continue to increase, above the rate of budgeted funding increases, due to demographic changes such as an ageing population, rising public expectations and medical advances [5];

· The NHS - and, therefore, NHS nurse and midwifery workforce planning - is politicized. It is funded by government; political priorities and funding levels change, and actual funding will not necessarily match planning based estimates of need or demand for nurses [6];

· The public sector is a monopoly provider of pre-registration education for nurses and midwives - all courses and places are funded and provided in the public sector;

· The public sector is the dominant employer of nurses and midwives - about four in every five working in England are employed in the NHS; the remainder are scattered across a range of small employers in social care, nursing homes and private sector hospitals [1].

These last three points highlight that government in England has more policy levers available than is the case in many other countries. Through decisions it makes on public sector funding, it determines the number of pre-registration places commissioned and funded in universities and other educational institutions; as the major employer (NHS) it dominates the nursing and midwifery labour market and sets the market rate for wages and employment/career benefits; and it also controls the inflow of nurses and midwives from other countries through migration policies [7].

## Methods

This paper analyses data on the broader nursing workforce in England drawn from sources in the public domain. A full list of sources is described in Additional file 1: Annex 1. The paper then uses scenario modelling to examine projections over the next ten years on the size of the NHS nursing workforce in England.

Scenario models provide a picture of what the future *might* look like under various assumptions. These outcomes can be quantified and the results used to assess the risks and opportunities of alternate policy decisions. Inevitably these models are a crude simplification, and their reliability is limited by the quality of the data available. Even with perfect data they can never be strictly “accurate” since they depend on the user to make judgements in specifying the future. In short, they are “projections” rather than “forecasts”.

The approach used in this paper is that of the aggregate deterministic supply model. It is aggregate in the sense that the workforce is treated as a group with common characteristics (e.g., occupation, age, gender). It is deterministic in the sense that stochastic variables (such as wastage) are treated as if the average value will occur each year without random variation. It is a supply model in that it simulates the interaction over time between the workforce (or “stock”), levels of demand for that workforce and the flows into, through and out of it. As such it can enable us to examine different strategies for balancing supply and demand over time.

Nevertheless, even with these simplifications, supply modelling in the labour market is difficult for two related reasons. First, the labour market is complex – nurses and midwives can move in many different ways between sectors, into and out of training and so on, throughout their careers. Second, the data on these stocks and flows are limited.

**Figure 1:**
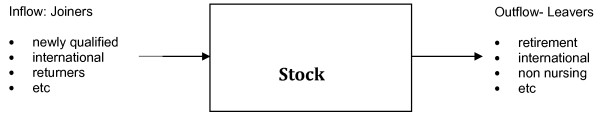
One-box stock-flow model.

The simplest kind of model represents the workforce as a single entity. This is known as a “one-box” model, and is the approach used for this paper (see Figure 1). The strength of supply models is in running them over a long period. The supply model used in this paper covered a ten year period.

### The current “stock”

The data required for the scenarios relates to the current “stock” working in the NHS in England, and to the various “flows” to and from this stock. The flows provide the dynamics within the system and they vary in magnitude across time. NHS nursing data are available as “headcount” and “whole time equivalent”. Headcount data were used for the modelling because of a lack of data on mobility expressed as whole time equivalents. Whole time equivalent data provide a better indication of the actual availability of nursing staff and are more appropriate as means of comparison across countries.

England is the largest of the four countries comprising the United Kingdom. Health policy is devolved to the four United Kingdom countries, but all have a similar NHS model, and all reported staffing growth across the period 2000 to 2010 [1]. This growth was driven by government investment in funding more nurse education places; implementation of policies to improve retention and return, and a commitment to a policy of active international recruitment up to 2006. These policy-led interventions and funding support had in turn been a response to recognized NHS nursing shortages in the late 1990s.

**Figure 2:**
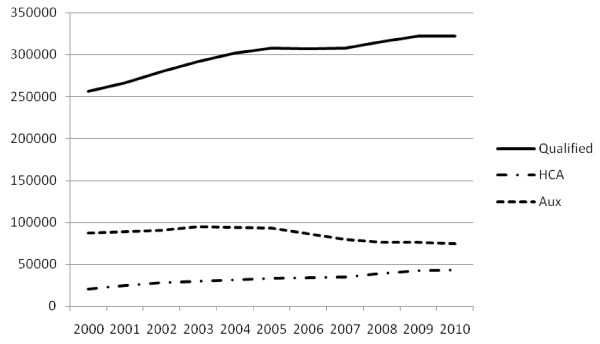
**NHS Staffing, England, 2000–2010: qualified nursing staff, nursing auxiliaries and healthcare assistants (HCA).** (Whole time equivalent).

NHS staffing trends in England across the last ten years are shown in Figure 2, which highlights that the growth in qualified nurses, midwives and health visitors tailed off after 2005, that the number of nursing auxiliaries (unqualified assistants) peaked in 2004 and has reduced year on year since, and there has been continued constant growth in the number of vocationally qualified health care assistants (HCA). Whole time equivalent data are used to provide an accurate comparison of availability across time.

At the time of the scenario modelling, the most recent NHS workforce data showed that there were 352 104 qualified (i.e., registered) nurses, midwives and health visitors in the NHS in England.

### Flows

The supply of “new” nurses and midwives to the NHS and to other employers in England comes mainly from pre-registration education in the country and, in some time periods, from international sources.

Supply from domestic pre-registration education training has been the major source in recent years. As noted earlier, pre-registration education is funded by government. Every year there are more applicants for nursing education in the United Kingdom than there are funded training places. Therefore, the number of student nurses entering pre-registration education in the country and subsequently entering the United Kingdom register when they qualify is not a random or uncontrolled event. It is the direct result of funding decisions by government and subsequent career choice by individuals.

**Figure 3:**
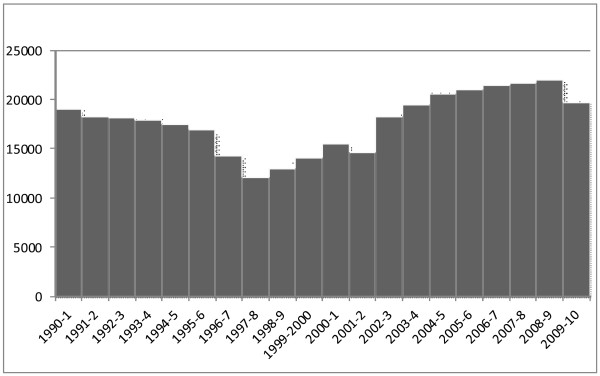
New entrants to the United Kingdom register from United Kingdom based pre-registration training 1990/1991 to 2009/2010.

The best measure available of inflow from United Kingom pre-registration is data from the Nursing and Midwifery Council (NMC) which presents annual data on the number of “new” nurses who register from United Kingdom based pre-registration education for the first time. A few individuals may complete their education and subsequently not register but the data source provides a good long term source of relevant data. An assessment of long term trends in intakes of “new” nurses and midwives entering the United Kingdom register from education and training in the United Kingdom shows that these reduced, year on year, in the early- to mid-1990s as the direct result of funding decisions to reduce the number of pre-registration places. The consequent drop in United Kingdom entrants was predictable, given decisions to reduce funding for pre-registration places, and was a major factor contributing to acknowledged nursing shortages later in the decade. Increased concern about nursing and midwifery shortages, and increased funding meant that there was a significant upward trend in intakes after 1997/1998 which led in turn to more “new” nurses coming out of pre-registration education (see Figure 3). This peaked in 2008/2009 and has subsequently dropped back as recent reductions in funding for intakes is beginning to have a knock on effect on new United Kingdom nurses entering the register [1].

As there is a lag of several years between decisions on funding levels for pre-registration nurse education and these “new” nurses and midwives entering the register, any funding related decisions for further reductions in intakes in the next few years will subsequently play through to training output declines, as happened in the mid 1990s.

In summary, historically large numbers of applicants are choosing to apply for pre-registration nursing programmes in England, but the actual funded intakes to training have been reducing in recent years. Looking forward, it is clear that the overall numbers of newly qualified nurses entering the labour market will fall as reductions in the number of places being commissioned feeds through into the numbers graduating; this has been compounded by the fact that the percentage of student nurses who fail to complete their studies appears to be significantly higher than planners have anticipated [8].

The second main source of “new” staff is those who have trained in other countries. International recruitment became an explicit policy solution for the NHS in England at the beginning of this century. The best source of data on international inflow and outflow is from the NMC. There are limitations in using NMC data to monitor the inflow of nurses and midwives to the United Kingdom, because it registers intent to move and work in the United Kingdom, rather than the actuality of working, but it is the best measure available. In the early part of the last decade, between 10 000 and 16 000 international nurses and midwives were added annually to the United Kingdom register - representing up to half of the total inflow in some years. This figure has now fallen to only 2500 per year, in 2010 [1]. International recruitment of these staff to the United Kingdom from non European Union (EU) countries has collapsed, in part because of reduced United Kingdom demand, and in part because migration policy changes have meant that entry to the United Kingdom for non EU nurses and midwives has become much more challenging and costly.

**Figure 4:**
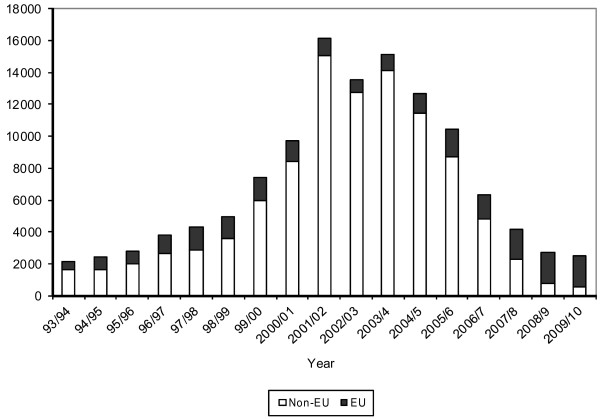
Admissions to the United Kingdom register from EU countries and other (non EU) countries 1993/1994 to 2009/2010.

The collapse in international recruitment of nurses and midwives is starkly obvious in Figure 4. NMC data give some sense of the massive pendulum swing in the number of international staff registering to practice in the United Kingdom. In the ten years between 1999/2000 and 2009/2010 the United Kingdom shifted from a low level of international recruitment activity in the late 1990s to very high levels of recruitment in the early part of this decade, then back down to low activity in recent years.

Outflow of nurses and midwives to other countries in 2009/2010 as measured by the number of verifications of qualifications issued by the NMC to other countries, was approximately 6300. In 2009/2010, 87% of all the verification requests from United Kingdom based nurses and midwives considering an international move were for just four destination countries: Australia, Canada, New Zealand and the United States of America – the main countries of the English speaking developed world [1].

### An ageing workforce

The ageing of the nursing and midwifery workforce is a major policy concern in many countries for two main reasons [9-11]. Firstly, older staff may exhibit different employment preferences and priorities than younger staff. For example, they may wish to reduce the hours they work, or avoid work that is too physically demanding, or may look to phase periods of work with other commitments. Secondly, as larger cohorts of staff move toward retirement age there are growing implications for replacement strategies. The policy challenge is to decide how the NHS will replace the skills and experience that it loses, as the large cohorts of staff that came into NHS employment in the 1970s and early 1980s retire from employment [12].

**Figure 5:**
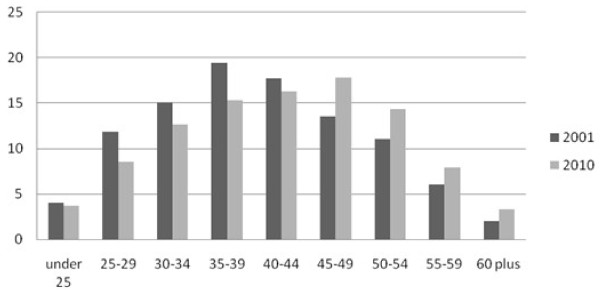
Age profile, United Kingdom Nurses and Midwives, 2001 and 2010.

The profession has been ageing consistently in recent years and this is reflected in ageing profiles of NHS nurses and midwives. The ageing of the NHS nursing workforce in England is shown in Figure 5. In 2001, about 19% of NHS England nurses were aged 50 or older; by 2010 this had increased to 26%. This overall age profile also masks big differences in different specialties and roles. For example, 36% of qualified nursing staff working in NHS England community services are 50 years old or older. The two much smaller groups of midwives and health visitors reported older age profiles than nurses.

The ageing of the workforce represents a major challenge to policy makers and planners, as a significant gap will be created when the nurses in older age cohorts retire from employment. This gap will become evident in the first instance in sectors that have older than average nursing workforce profiles, such as community nursing and nursing homes.

## Results

This section describes and examines different possible scenarios for the supply of NHS nurses in England over the ten year period to 2012/2022 which were used in the model. (Additional file 1: Annex I gives detailed evidence behind the assumptions).

As part of this exercise, eight scenarios were selected and modelled. These were:

A. “No change” - current inflows and outflows

B. “Redundancies” - current inflow with higher outflow

C. “Improved retention” - current inflow with lower outflow

D. “Reduced training intakes A” - lower inflows with lower outflow

E. “Reduced training intakes B” - lower inflow with higher outflows

F. “Pension time-bomb” - current inflow with a higher rate of retirement

G. “Pension delayed” - current inflow with a lower rate of retirement

H. “Worst case” - lower inflow and higher outflow including higher retirement

The starting stock for all the scenarios was the most recent NHS workforce census data which showed a headcount figure of 352 104 for qualified (i.e., registered) nurses, midwives and health visitors in England as of September 2010; this was used as the 2011/2012 baseline. The model uses headcount figures. Although part-time working is significant in nursing, data on flows of full time equivalent numbers (from education into the workforce and leaving the workforce) are not available.

Table 1 summarizes the key results of each of the eight scenarios, which are discussed below.

**Table 1:** Summary model outcomes

**Scenario**		**NHS England: Staff in post 2021/22**	**Change on 2010/11**	**% change**
		**(headcount)**		
A	Current rates	309 297	−42 807	−12.2%
B	Current inflow & higher outflow	296 083	−56 021	−15.9%
C	Current inflow & lower outflow	385 723	+33 619	+9.5%
D	Lower inflow & lower outflow	358 734	+6 630	+1.9%
E	Lower inflow & higher outflow	271 177	−80 927	−23.0%
F	Current inflow, outflow & higher retirement	290 783	−61 321	−17.4%
G	Current inflow, outflow & lower retirement	342 844	−9 260	−2.6%
H	Lower inflow, higher outflow & higher retirement	253 088	−99 000	−28.0%

Source; [1].

Scenario A represents a “steady state” model. It projects forward staff numbers using the best available recent intake and outflow estimates. The “steady state” assumes no other changes in assumptions on factors affecting inflow and outflow. Using these estimates the model shows that the NHS nursing workforce would shrink by just over 1% a year. By 2021/2022, the model predicts an NHS nursing workforce of about 309 300. This is a projected decline of 12% (42 800) over the next ten years.

Scenario B assumes higher rates of outflow (leavers, other than retirements, increase to 6.5%). This could occur if retention decreased and/or retirement increased. Historically high levels of actual outflow were used as an indicator. The effect is a fall of roughly 16% in the NHS nursing workforce over the period. By 2021/2022, the model projects an NHS nursing workforce of 296 000, some 56 000 lower than at the start of the projection.

Scenario C assumes lower rates of outflows (leavers, other than retirements, falls to 3.5%). This could occur if retention of staff improved - either as a result of active policies, or because there were fewer non-NHS nursing opportunities for employment. The effect is enough to change the forecast from a deficit into a small increase. By 2021/2022, the model projects an NHS nursing workforce of about 385 700, more than 9% (33 600) higher than at the start of the projection.

Scenario D combines the lower outflow of scenario C with smaller inflows from education. The smaller inflows from education were determined using historical data on trends in reduction in intakes to pre-registration nurse education that occurred in the 1990s. Initially, the lower outflow means that the workforce grows slightly. However, from 2016/2017 onwards, as lower student intakes start to take effect, the workforce reduces. Overall these effects balance each other out so that by 2021/2022 the workforce is marginally (1.9% or 6600) larger.

Scenario E combines the lower intakes of scenario D with higher outflows (other than retirement). This combination of variables generates a very large reduction (almost 81 000) in the nursing workforce within ten years. This produces an NHS nursing workforce of 271 200 by 2021/2022, some 23% smaller than now.

Scenario F combines current inflows and outflows with a higher rate of retirement. Increased retirement could occur if more nurses decide to voluntarily leave NHS employment at an earlier age, perhaps because of job stress or workload. As a consequence, inflows cannot match the outflows and the overall nursing workforce reduces by just over 61 000, losing one in six nurses currently employed. By 2021/2022 the workforce would be around 290 800.

Scenario G combines current inflows and outflows with delayed retirement. Delayed retirement can occur when individual staff chose to work on until an older age (e.g., if they need the additional financial contributions and pension entitlement) or when there is an increase in the mandatory retirement age. Unlike the previous scenario this produces a comparatively small reduction, of 9260 (2.6%) and a workforce in 2021/2022 of 342 850.

Scenario H represents the “worst case” in which smaller inflows combine with higher outflows and a faster rate of retirement to shrink the workforce very rapidly. Over ten years more than a quarter of the nursing workforce (99 000) would be lost leaving staff in post of just 253 000 in 2021/2022.

**Figure 6:**
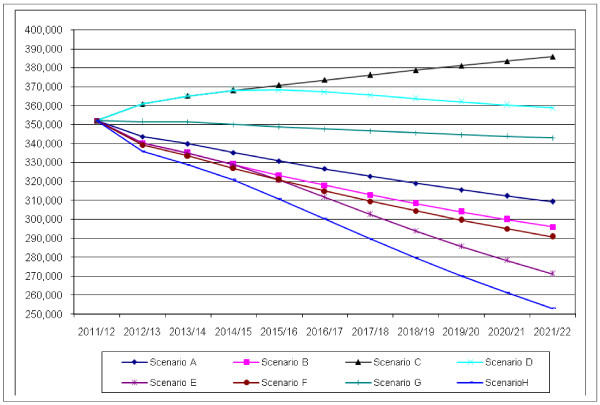
Projected NHS qualified workforce in England 2011/2012 to 2021/2022.

Figure 6 highlights the results of the eight different scenarios. It shows that under most scenarios there would be a reduction in NHS staffing numbers over the ten year period and also illustrates the overall “best” and “worst” case scenarios for future supply.

## Discussion

The scenarios demonstrate the effect of any changes in the relative size of new intakes to pre-registration education and of changes in the retention of existing staff, in determining NHS staff supply over the period to 2022.

In relation to intakes to pre-registration education, the scenarios are developed from a situation in which there have been marked fluctuations in the numbers of student nurses and midwives being trained over the last 20 years, with a sense of “boom and bust”, and with a recent trend towards reduced funding for intakes. Combined with the fact that there has been little evidence of sustained improvement in student nurse attrition rates in recent years, this suggests that it is likely that there will be a sustained reduction in the size of new inflow of nurses to the NHS over the foreseeable future.

In relation to retention, the ageing profile of the workforce has been much discussed in recent years, but the NHS is now entering a period when ageing and retirement patterns will become critical factors. Even with delayed retirement for some nurses and midwives, many more of these NHS staff will soon be reaching the age when they consider retirement or working reduced hours. Relatively small overall changes in the percent of nurses and midwives deciding to retire in any given year will have an increasing impact because relatively more nurses will be in the older, pre-retirement cohorts.

Any scope for improved retention will have to be based on policies that meet the employment and career needs of older staff as well as being sufficiently flexible to meet changing priorities should the United Kingdom move into a stronger economic situation and labour markets tighten.

**Figure 7:**
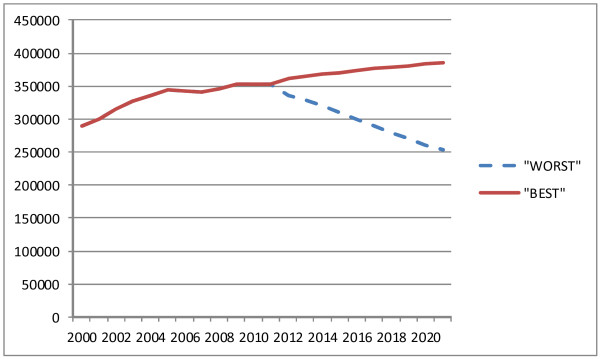
Trend in supply of NHS staff, England (2000–2010), and “best” and “worst” case scenarios 2011–2022 (headcount).

Most of the scenarios indicate that a reduction in the supply of staff to NHS England is possible over the next ten years. This would mark a reversal of trends in the last ten years. Putting the “best” and “worst” case scenarios in the context of longer term trends in NHS nursing supply (Figure 7) highlights that the overall pattern has shifted from relatively rapid growth in numbers in the period from 2000 to 2005, followed by a tailing off in growth between 2006 and 2010, and with a possibility of growth being replaced by staffing reductions between 2011 and 2020.

Policy makers will have to assess the range of possibilities set out in the scenarios, understand the implications of their policy decisions, and take responsibility for policy action related to funding new intakes to pre-registration and retention of current staff.

These scenarios also have significant implications for decisions on overall skill mix in the NHS. The numbers of registered nurses and midwives must be examined within the context of the numbers of other health workers in the overall workforce. As highlighted earlier, the main recent area of staffing growth has been continued increase in the number of Health Care Assistants (HCAs). A continuation of this trend, allied to any significant downward shift in the numbers of NHS nurses will intensify the debate about the appropriate skill mix deployed in various care environments in the NHS in England. Policy makers will have to be confident that staffing mix, locally and nationally, are based on a rational assessment of workload, quality and appropriate skills, rather than being driven only by any changes in supply.

## Conclusions

The scenarios examined in this paper highlight the vulnerability of the size of the NHS workforce in England to policy changes, particularly in terms of the numbers of pre-registration education places being commissioned and the impact of changes to retirement policies.

The projections illustrate that small changes in assumptions can make a substantial difference to outcomes and therefore emphasize the point that it is unwise to base policy decisions on a single projection. It is important that different scenarios are considered that may be regarded as possible futures, based on a realistic assessment of the available workforce data, policies and broader labour market and funding outlook.

The main limitation in the scenario approach is that it is dependent on the availability of relevant data. While no system has “perfect” workforce data, there are sufficient data available in the NHS in England to develop an approach with acceptable levels of accuracy and completeness. It is also critical that the assumptions made in using the data and developing the scenarios are within realistic bounds. The approach detailed in this paper is based on a careful analysis of long term trends and current policy priorities and constraints. It does not claim to make any predictions about the future size and shape of the NHS workforce - it uses scenarios to make projections based on the best current data availability. As such, using the most recent data and a variety of assumptions, the paper illustrates that there is a strong risk that current policy decisions could lead to a significant reduction in NHS staffing numbers in England over the period to 2022.

## Competing interests

The authors declare that they have no competing interests.

## Authors’ contribution

JB and IS developed the overall concept and design, identified data sources and developed scenarios. IS conducted the modeling. JB and IS drafted the paper. Both authors read and approved the final manuscript.

## Supplementary Material

Additional file 1: Annex 1 Estimates on Inflows and Outflows. Click here for file
