# Supplementary material for: Using scenarios to assess the future supply of NHS nursing staff in England
Source: Hum Resour Health. 2012 Jul 12;10:16. doi: 10.1186/1478-4491-10-16 (PMC3459709; doi:10.1186/1478-4491-10-16)
Supplement: Additional file 1: Annex 1 — Estimates on Inflows and Outflows. [file 1478-4491-10-16-S1.docx]

**Annex 1: Estimates on Inflows and Outflows**

Full information on the data, data sources and estimates used in developing the scenarios can be found at Buchan and Seccombe, 2011.

http://www.rcn.org.uk/__data/assets/pdf_file/0006/405483/LMR2011_FINAL.pdf

The two main sources of primary data used to inform the work are:

1)The workforce census data published by the Information Centre

<http://www.ic.nhs.uk/statistics-and-data-collections/workforce/nhs-staff-numbers/nhs-staff-2000--2010-non-medical>

2) The Nursing and Midwifery Council

[www.nmc-uk.org](http://www.nmc-uk.org)

**Inflows**

As discussed in the text , the main sources of inflows are new qualifiers, re-entrants to the workforce after a period of absence and net in-migration .

*Inflows – annual intakes to pre-registration nurse training*

• Planned commissions in England for nursing and midwifery in 2011/12 are currently estimated at 20,495

• Annual intakes to education are assumed to remain level for the next 10 years for Scenario A (no change), Scenario B (redundancies), Scenario C (improved retention), Scenario F (pension time-bomb) and Scenario G (pension delayed).

• Scenarios D and E (reduced training intakes) and Scenario H (worst case) examine the impact of planned commissioned falling below current levels.

• Using figures obtained from the Nursing Standard from higher education institutions, we assume an attrition rate of 28%.

• Not all nurses who qualify will go on to work in the NHS. By comparing the number of new qualified nurses aged under 25 in NHS employment (13,040 in September 2010 non-medical workforce census) with the number on the NMC professional register (15,200 as at March 2010) we assume that 85% of all newly qualified nurses were working in the NHS in 2010. This is a crude figure but similar to other estimates. Applying the 85% figure suggests that from a commissioning intake of 20,495 the NHS can expect to recruit around 12,540 nurses and midwives three years later.

• Historical attrition rates of 28% and NHS participation rates of 85% are maintained.

• From 2015/16 onwards, the number of new qualifiers entering NHS employment is fixed in Scenario A (no change), Scenario B (redundancies), Scenario C (improved retention), Scenario F (pension time-bomb) and Scenario G (pension delayed).

• Scenarios D and E (reduced training intakes) and Scenario H (worst case) assume further reductions in places commissioned and subsequent inflow to employment of new qualifiers. These three scenarios assume an intake reduction of 11% each year for three years. This figure is based on the actual reduction seen in the last major downturn in numbers commissioned (31% between 1991/92 to 1994/95). This scale of reduction would mean the number of intakes falling from 18,445 in 2012/13 to 14,490 in 2014/15. Assuming no change in attrition rates, the number entering NHS employment from 2017/18 would be just under 8,000 (compared with 12,450 now).

*Inflows – International nurses*

• As noted earlier, the international inflow of nurses to the UK has recently fallen due to a combination of reduced demand and stricter entry restrictions for nurses from non-EU countries.

• There were 2,000 initial entrants to the NMC professional register from EU countries in 2009/10 plus 550 from non-EU countries.

• We assume that 95% work in England and that 85% of these work in the NHS and this is a constant inflow in all scenarios.

*Inflows – other labour market entrants*

• We assume an annual inflow of 8,000 of nurses within the UK – people returning to practice after a break or from living abroad or new qualifiers who delayed their entry to the NHS. This is based on 28,348 new joiners among the nursing workforce, and taking out estimates of newly qualified joiners (18,000) and international inflow (around 2,000).

• This figure is kept constant for all scenarios, but we acknowledge the possibility of a ‘recession benefit’ to the nursing labour market in the short term, as more nurses come back into employment or increase working hours.

**Outflows**

The main destinations of **outflow or losses from the workforce** include retirements, resignations, redundancies, and out-migration.

*Outflows – retirement*

• Based on recent trends, likely patterns for retirement for the next 10 years are for all nursing staff aged over 55 and around 40% of those aged 50-55 to retire in the next 10 years (a total of 40,722).

• The Workforce Review Team’s modelling work predicts that the retirement rate will rise from 1.6% to 2.9% over 2011 to 2020.

• For Scenarios A to E, we assume that the retirement rate remains constant.

• For Scenario F (pension time-bomb) and Scenario H (worst case), we assume that all those currently aged 50 and over will retire in the next 10 years.

• For Scenario G (lower retirement) we assume that only those aged 55 and over will retire.

*Outflows – other leavers*

• Data from the NHS Information Centre shows that 28,697 nurses, midwives and health visitors left the NHS in England between January 2010 and January 2011 – an outflow of around 8.2%.

• Removing the number accounted for by retirement outflows leaves around 22,000 other leavers or 6%.

• This 6% figure is kept as a constant in Scenario A (no change).

• We assume it increases to 6.5% in Scenario B (redundancies), Scenario E (reduced training intake) and Scenario H (worst case). The 6.5% is within the range recorded by Office for Manpower Economics surveys conducted for the NHS Pay Review Body.

• We assume this figure to fall to 3.5% for Scenario C (improved retention), and Scenario D (reduced training intake) due to improved retention rates. The 3.5% figure is used by the WRT for the years 2009 to 2020 in its model of the nursing labour market.
